# The contribution of health policy and care to income differences in life expectancy – a register based cohort study

**DOI:** 10.1186/1471-2458-13-812

**Published:** 2013-09-08

**Authors:** Kristiina Manderbacka, Riina Peltonen, Sonja Lumme, Ilmo Keskimäki, Lasse Tarkiainen, Pekka Martikainen

**Affiliations:** 1 Service System Research Unit, National Institute for Health and Welfare, P.O. Box 30, Helsinki 00271, Finland; 2 Department of Social Research, Population Research Unit, University of Helsinki, P.O. Box 54, Helsinki 00014, Finland; 3 Service System Department, National Institute for Health and Welfare, P.O. Box 30, Helsinki 00271, Finland; 4 School of Health Sciences, University of Tampere, Tampere 33014, Finland

**Keywords:** Mortality, Life expectancy, Socioeconomic factors, Amenable mortality, Equity research

## Abstract

**Background:**

Growing mortality differences between socioeconomic groups have been reported in both Finland and elsewhere. While health behaviours and other lifestyle factors are important in contributing to health differences, some researchers have suggested that some of the mortality differences attributable to lifestyle factors could be preventable by health policy measures and that health care may play a role. It has also been suggested that its role is increasing due to better results in disease prevention, improved diagnostic tools and treatment methods. This study aimed to assess the impact of mortality amenable to health policy and health care on increasing income disparities in life expectancy in 1996-2007 in Finland.

**Methods:**

The study data were based on an 11% random sample of Finnish residents in 1988–2007 obtained from individually linked cause of death and population registries and an oversample of deaths. We examined differences in life expectancy at age 35 (e_35_) in Finland. We calculated e_35_ for periods 1996-97 and 2006-07 by income decile and gender. Differences in life expectancies and change in them between the richest and the poorest deciles were decomposed by cause of death group.

**Results:**

Overall, the difference in e_35_ between the extreme income deciles was 11.6 years among men and 4.2 years among women in 2006-07. Together, mortality amenable to health policy and care and ischaemic heart disease mortality contributed up to two thirds to socioeconomic differences. Socioeconomic differences increased from 1996-97 by 3.4 years among men and 1.7 years among women. The main contributor to changes was mortality amenable through health policy measures, mainly alcohol related mortality, but also conditions amenable through health care, ischaemic heart disease among men and other diseases contributed to the increase of the differences.

**Conclusions:**

The results underline the importance of active health policy and health care measures in tackling socioeconomic health inequalities.

## Background

Growing mortality differences between socioeconomic groups have been reported both in Finland and other developed countries during the last decades [1-3]. In a recent study from Finland, Tarkiainen et al. [4] report growing income group differences in life expectancy from the late 1980s to 2007. The gap in life expectancy at age 35 between the highest and lowest income quintile widened by 5.1 years among men and 2.9 years among women from 1988-92 to 2003-07. At the end of the study period they reported a 12.5 year difference among men and a 6.8 year difference among women. The most important causes of death contributing to these differences were alcohol related deaths and some cancers in both genders and ischaemic heart disease (IHD) among men [4]. Socioeconomic differences in health behaviours have been reported in Finland and they have been estimated to explain some of the differences in IHD morbidity and to contribute to differences in IHD and all-cause mortality in Finland [5,6] and elsewhere [7]. While health behaviours and other lifestyle factors are important in contributing to health differences, some researchers have suggested that some of the mortality differences attributable to lifestyle factors, mainly tobacco and alcohol related causes, could be preventable by health policy measures including, e.g., health promotion measures and price policies [8,9].

It has also been suggested that health care may play a role, and that its role is increasing due to better results in disease prevention, improved diagnostic tools and treatment methods, and rehabilitation especially in conditions such as stroke, IHD and some cancers [10]. While health care has a possibility to decrease health differences between socioeconomic groups, earlier research from Finland and elsewhere on health care use suggests that this might not be the case but, instead, care has been reported to be distributed pro-rich in different parts of the service chain in relation to need [11-15].

Mortality amenable to health care refers to unnecessary and premature deaths that should not occur in the presence of timely and effective health care. It has been increasingly used as an indirect measure of health care performance. While mortality amenable to health care has declined in all Western countries during the last decades [16], it has nevertheless been estimated to contribute ca 20 per cent to total mortality under the age of 75 in Western countries [17]. Earlier research from European countries [18], New Zealand [19], Australia [20], Canada [21] and the U.S. [22] suggests a consistent pattern with those at risk of social disadvantage being also at higher risk of death from causes amenable to health care irrespective of the definition of amenable mortality or age limits used. An early study by Mackenbach et al. [23] suggests that medical care can have had an impact on widening of total mortality differentials between socio-economic groups between 1931 and 1981 in England and Wales and between 1952 and 1982 in The Netherlands. The results of more recent studies have been mixed: both decreasing [21,24], stable [25] and increasing [20] socioeconomic differences have been reported in mortality amenable to health care from different countries. A recent study from Finland shows increasing inequity in relative differences in total mortality amenable to health care and especially in mortality amenable to action in specialized health care. Absolute differences between income groups remained substantial and stable [26]. Earlier research has not studied the contribution of mortality amenable to health care interventions to socioeconomic differences in total mortality or changes in them.

The aim of the study is to examine the contribution of mortality amenable to health policy and health care interventions to socioeconomic differences in life expectancy at age 35 in Finland. We analyse the contribution of mortality amenable to health care, two types of causes related to health behaviours and amenable through health policy measures and IHD mortality to income differences in mortality and compare their contribution to that of other causes of death. In addition we analyse the contribution of mortality amenable to health policy and health care to changes in income disparities in life expectancy. A methodological aim of the study is to analyse socioeconomic differences in partial life expectancy (between 35 and 75 years), since 75 is usually used as an upper limit when examining amenable mortality. We use income as an indicator of socioeconomic position, since it is more sensitive to changes in individuals’ circumstances than education or occupational social class.

## Methods

### Data, study population and period

The study was based on a register derived data that was formed in Statistics Finland by drawing an 11 per cent random sample of all the individuals residing in Finland in at least one of the years between 1988 and 2007 and an oversample of those who died during the years so that altogether 80 per cent of all deaths between the years 1988 and 2007 were included. In the data the individuals included are followed up annually. Statistics Finland used personal identification codes to link information from different registers and cause of death records. Weights for each individual were constructed using known sampling probabilities in order to take into account the sampling design. The oversample of deaths increases the statistical power of our analyses.

From this data described we included all the non-institutionalised persons aged 35 or more at the end of the years 1995, 1996, 2005 or 2006. These individuals were followed up annually during 1996-1997 and 2006-2007. Age and income were measured yearly on the basis of information from the year preceding each follow up year. Follow up times of those who died were cut to date of death and time spent emigrated was excluded. We then aggregated person years and deaths by gender, study period (1996-1997 and 2006-2007), income group and age group. Altogether, there were 61,515 deaths in 1996-1997, and 60,422 deaths in 2006-2007 in the unweighted data.

### Income measure

Our income measure is based on the information provided by the Finnish tax administration and the Social Insurance Institution. We used household disposable income that combines all taxable income and non-taxable income transfers of those living in the same household and excludes taxes. To account for differences in household size and composition income was divided by the sum of consumption units in the household using the OECD modified equivalence scale which assigns the weight of 1 unit for the first adult, 0.5 units for other household members aged more than 13 years and 0.3 units for children aged 13 or less [27]. Income deciles were constructed for each year on the basis of information at the end of the preceding year and cut-off points calculated from income information of the whole population aged 35 and over at the measurement point. While earlier research has examined differences between income quintiles, we used income deciles in order to better detect the effects of income poverty to life expectancy. Sensitivity analysis was conducted for 2006-07 with income deciles constructed at the end of 2003 and 2004.

### Cause of death grouping

We studied alcohol related and lung cancer mortality as causes of death that can be influenced by health policy measures [8,9,28], mainly by price policy and limitations in the availability of alcohol and tobacco products. These causes of death were defined using the classification of diseases by Statistics Finland. Alcohol related deaths were defined as those in which alcohol was selected as the underlying cause of death taking into account not only deaths caused directly by alcohol but also diseases caused by excess alcohol consumption [29]. Additional file 1 presents the classification with the relevant ICD-codes (Additional file 1).

While the conditions selected to represent mortality amenable to health care vary, those in which a specific health care intervention can be defined are usually chosen. We used the list of causes of death summarized by Nolte and McKee [10] from a systematic review and supplemented it with some conditions from the Australian and New Zealand Atlas of Amenable Mortality [9]. We further used a sub-categorization of amenable mortality modified from Simonato et al. [8] to indicate the main place of the potentially effective intervention to one more suitable to Finnish health care system: specialized health care and primary health care. This grouping is somewhat arbitrary but has analytic and descriptive value in pointing out the sectors where weaknesses in the care system should be analysed further. The list of conditions used in defining amenable mortality is presented in Additional file 1. We omitted deaths related to “misadventures in health care”, since Statistics Finland does not report them separately in the Cause of death statistics. Additionally, alcohol related epilepsy mortality was omitted from causes amenable to health care interventions since it was included in the Statistics Finland classification of alcohol related causes.

In Finland, general practice level ambulatory services are primarily provided by the public sector health centres and financed through taxation. These services have low co-payments for patients but there have been problems in access. In addition, private ambulatory services are available especially in cities and large municipalities, but patients’ co-payments are high. Occupational health services provide easy access to ambulatory care for employed persons free of charge. The municipalities form 20 hospital districts, which organise and provide specialist medical services for the residents and are managed and funded by the municipalities [30]. While general practitioners act as gate-keepers for public specialist outpatient and inpatient services in the public sector and in occupational health care, no gate-keeping is exercised in private services.

It has been suggested, that part of IHD mortality should also be included in conditions amenable to health care [10] and part of IHD mortality is related to lifestyle factors that could be influenced through policy interventions [31-34]. Consequently, we included IHD deaths to the analyses as a separate category, since it is not evident, how deaths due to IHD should be allocated in these categories of amenable mortality. Mortality from other causes comprise of all causes not included in the described categories.

### Methods

We examined two age spans: life expectancy at age 35 (e_35_) and partial life expectancy between 35 and 75 years of age (e_35-75_). Partial life expectancy was included in the analyses, since mortality amenable to health care is usually defined as mortality under the age of 75. We calculated both life expectancies using an abridged life table [35] with age-specific mortality rates aggregated from the individual level data in 5-year age groups for 1996-97 and 2006-07 by income decile and gender. We then decomposed the difference in life expectancies between the highest and the lowest decile in 1996-97 and 2006-07 by cause of death grouping (mortality amenable to policy measures, mortality amenable to health care interventions, IHD, other causes) [36-38]. We also decomposed the change in life expectancy in the extreme deciles from 1996-97 to 2006-07 by four cause of death groups (and relevant subcategories) and examined how differential development in cause specific mortality in these deciles contributed to change in the life expectancy difference between the deciles. In the decomposition of life expectancy at age 35 the amenable mortality categories were only considered to be amenable under the age of 75.

## Results

Table 1 shows differences in life expectancies between income deciles in both time periods. Both life expectancies show a linear gradient from the highest to the lowest decile among both men and women. The difference in life expectancy at 35 between the highest and lowest income decile was 11.6 years among men and 4.2 years among women in 2006-07. For partial life expectancy from 35 to 75 years, the difference for men was 7.2 years and for women 2.3 years. While both life expectancies increased in most income deciles among both genders from 1996-97 to 2006-07, we found a decrease in two lowest deciles among men for life expectancy at 35 and among both genders for partial life expectancy between 35 and 75.

**Table 1:** Life expectancies (e35 and e35-75) by income decile, men and women in 1996-1997 and 2006-2007

	**Men**				**Women**			
	**1996-1997**		**2006-2007**		**1996-1997**		**2006-2007**	
**Income decile**	**Life expectancy**	**(95% Cl)**	**Life expectancy**	**(95% Cl)**	**Life expectancy**	**(95% Cl)**	**Life expectancy**	**(95% Cl)**
e35								
1. (highest)	44.4	(43.4–45.4)	47.1	(46.2–48.0)	49.1	(48.0–50.1)	51.4	(50.4–52.5)
2.	43.1	(42.2–44.0)	46.7	(45.8–47.7)	48.1	(47.1–49.2)	51.0	(49.9–52.1)
3.	42.0	(41.1–42.8)	45.6	(44.8–47.7)	47.1	(46.8–48.6)	50.6	(49.6–51.5)
4.	40.9	(40.2–41.7)	44.8	(44.0–45.6)	47.2	(46.4–48.0)	50.1	(49.2–51.0)
5.	40.7	(40.0–41.4)	44.2	(43.5–45.0)	47.0	(46.3–47.7)	49.7	(49.0–50.2)
6.	39.7	(39.0–40.4)	43.2	(42.5–43.9)	46.8	(46.1–47.5)	49.5	(48.8–50.2)
7.	38.9	(38.2–39.6)	41.4	(40.7–42.1)	46.0	(45.3–46.7)	48.9	(48.3–49.6)
8.	37.4	(36.6–38.2)	39.5	(38.7–40.3)	45.6	(44.9–46.3)	47.4	(46.7–48.1)
9.	36.4	(35.6–37.3)	36.2	35.3–37.1	45.6	(44.9–46.4)	46.4	(45.7–47.2)
10.	36.2	(35.3–37.0)	35.6	(34.7–36.4)	46.6	(45.9–47.3)	47.3	(46.5–48.0)
Difference (1.–10.)	8.2		11.6		2.5		4.2	
e35-75								
1. (highest)	37. 0	(36.7–37.3)	38.0	(37.7–38.2)	38.4	(38.1–38.7)	38.8	(38.6–39.1)
2.	36.6	(36.2–36.9)	37.8	(37.5–38.1)	38.1	(37.8–38.4)	38.8	(38.6–39.0)
3.	36.0	(35.7–36.4)	37.4	(37.2–37.7)	38.1	(37.9–38.4)	38.7	(38.5–38.9)
4.	35.6	(35.2–36.0)	37.0	(36.7–37.3)	37.9	(37.7–38.2)	38.4	(38.2–38.7)
5.	35.4	(35.0–35.8)	36.7	(36.4–37.0)	38.0	(37.7–38.2)	38.4	(38.2–38.7)
6.	34.8	(34.4–35.3)	36.1	(35.7–36.5)	37.7	(37.4–38.0)	38.1	(37.9–38.4)
7.	34.3	(33.8–34.8)	35.0	(34.5–35.5)	37.3	(37.0–37.7)	37.9	(37.6–38.2)
8.	33.0	(32.4–33.5)	33.8	(33.3–34.3)	36.9	(36.5–37.3)	37.0	(36.7–37.4)
9.	32.0	(31.4–32.6)	31.4	(30.8–32.1)	36.7	(36.3–37.2)	36.4	(35.9–36.8)
10. (lowest)	31.8	(31.2–32.3)	30.8	(30.3–31.4)	37.10	(36.6–37.5)	36.5	(36.1–37.0)
Difference (1.–10.)	5.3		7.1		1.3		2.3	

e35 = Total life expectancy at the age 35.

e35-75 = Partial life expectancy between ages 35 and 75.

Table 2 presents the decomposition of the differences in life expectancy at age 35 and partial life expectancy from age 35 to 75 in 2006-2007. Mortality amenable to health policy measures (deaths from alcohol related causes and lung cancer) contributed 35 per cent (4 years) to socioeconomic differences in life expectancy at age 35. Overall, alcohol related deaths made the most significant contribution to the life expectancy differences at age 35 among men. Mortality amenable to health care interventions contributed nine per cent (1 year) and IHD mortality 19 per cent (2.3 years) in 2006-07. Other causes contributed by over a third to the difference. As for men, mortality amenable to health policy measures contributed to the difference by almost a third (1.2 years) among women in 2006-07. This was mainly attributable to alcohol related mortality (1.1 years, 25%), which made a large contribution to mortality differences for women. Lung cancer mortality made a contribution of approximately four per cent. Mortality amenable to health care contributed 17 per cent (0.7 years) to the total difference between the highest and lowest income deciles. This was mainly attributable to mortality amenable through primary care. Differences in IHD mortality were responsible of one fifth of the gap (0.9 years). Other causes contributed to the difference by approximately one third.

**Table 2:** Contribution of mortality difference to differences in life expectancy between the extreme deciles in 2006-2007

			**Total difference in LE**		**Deaths amenable to health policy and care**							
					**Deaths amenable to health policy**			**Deaths amenable to health care interventions**				
				**Total**	**Total**	**Alcohol related**	**Lung cancer**	**Total**	**Primary care**	**Specialised care**	**IHD**	**Other causes**
e35	Men	Years	11.56	7.29	4.00	3.40	0.60	1.04	0.72	0.32	2.25	4.26
		Per cent	100.00	63.13	34.68	29.45	5.23	9.01	6.21	2.80	19.44	36.87
	Women	Years	4.18	2.83	1.22	1.05	0.17	0.71	0.62	0.08	0.90	1.34
		Per cent	100.00	67.88	29.33	25.15	4.18	16.94	14.93	2.01	21.61	32.10
e35-75	Men	Years	7.15	4.29	2.61	2.34	0.27	0.63	0.43	0.20	1.05	2.85
		Per cent	100.00	60.10	36.58	32.79	3.79	8.87	6.07	2.81	14.65	39.89
	Women	Years	2.33	1.39	0.75	0.66	0.09	0.40	0.34	0.06	0.24	0.94
		Per cent	100.00	59.65	32.28	28.51	3.77	17.25	14.68	2.57	10.12	40.36

More than seven years difference was found in life expectancy from age 35 to 75 in 2006-07 among men and 2.3 among women (Table 2). In this group, mortality amenable to health policy measures or health care together with IHD mortality accounted for about 60 per cent of the income group difference between the highest and the lowest decile. The main contribution to the difference was made by alcohol related deaths and other cause deaths among men, and alcohol related deaths, other cause deaths and mortality amenable to health care among women. For this age group the contribution of mortality amenable to health care to the difference in life expectancy in women was larger than that of IHD mortality.

**Figure 1:**
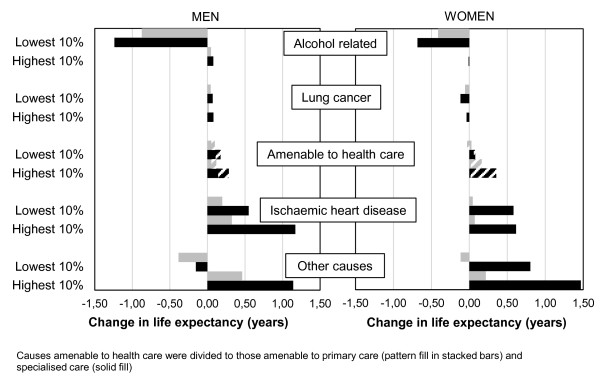
**The contribution of cause of death groups to change in life expectancy by income.** The contribution of cause of death groups to change in life expectancy from 1996-1997 to 2006-2007 in the highest and lowest income decile among men and women. Causes amenable to health care were divided to those amenable to primary care (pattern fill in stacked bars) and specialised care (solid fill).

Figure 1 shows the contribution of the examined cause of death groups to change in the life expectancies (e_35_ and e_35-75_) from 1996-97 to 2006-07 in the extreme deciles. Among men life expectancy at 35 decreased by 0.6 years in the lowest income decile and increased by 2.8 years in the highest. The single most important contributor to the increase in differences in life expectancy was deaths amenable through policy measures, mainly alcohol related mortality, which declined somewhat in the highest income decile and showed a large increase in the lowest decile contributing 1.2 years to decline of their life expectancy. While IHD mortality decreased in both of the extreme deciles, the decrease was larger in the highest decile. Decline in mortality amenable to health care, mainly primary health care, was also larger in the highest decile. Among women, life expectancy at 35 increased by 2.4 years in the highest and 0.7 years in the lowest decile. Decline in both IHD and other cause mortality was an important contributor to the increase in life expectancy in both extreme deciles, but in other cause mortality the decline was larger in the highest decile. Additionally, mortality amenable to health care, mainly primary health care, decreased more in the highest decile and mortality amenable to health policy measures, more specifically alcohol related mortality, increased in the lowest decile contributing 0.7 years to decrease of their life expectancy.

The difference in partial life expectancy (35-75) between the extreme deciles increased by 1.9 years among men and about one year among women from 1996-97 to 2006-07. Among men the partial life expectancy decreased by 0.9 years in the lowest income decile and increased by almost one year in the highest. As in life expectancy at 35, differential development in mortality amenable through policy measures, namely alcohol related mortality was the most important contributor to the increase of the differences. Additionally, other cause mortality showed differential development in the extreme deciles. Among women in the lowest decile, an increase was detected in all causes of death groups except mortality amenable to specialised care and IHD mortality. In the highest decile all causes of death groups contributed to the increase of life expectancy. As for life expectancy at 35, differential development was detected in mortality amenable through policy measures. While alcohol related mortality did not change in the highest decile, a large increase was detected in the lowest decile. In addition, while other cause mortality decreased in the highest decile, it increased in the lowest decile. Mortality amenable to (primary) health care also increased slightly in the lowest income decile but decreased in the highest.

## Discussion

Our study examined the contribution of health policy and health care to income differences in life expectancy at age 35 and partial life expectancy from age 35 to age 75 in 2006-07 and to changes in them from 1996-97 to 2006-07. Mortality amenable to health policy measures, mainly alcohol related deaths, accounted for a large part of the differences in life expectancy between top and bottom deciles at age 35 among both men and women in 2006-2007. Ischaemic heart disease mortality accounted for about 20 per cent of the differences among both genders, and the contribution of mortality amenable to health care was also important especially among women (17%).

According to our results income differences in life expectancy at age 35 increased from 1996-97 to 2006-07 by 3.4 years among men and 1.7 years among women. Further, while life expectancy increased by 2.8 years among men in the highest income decile, life expectancy did not increase in the lowest decile, but decreased by 0.6 years. In line with earlier research suggesting increasing contribution of life style related causes [21] differential development in alcohol related mortality and larger decrease in IHD mortality in the highest income decile were important contributors to increase of socioeconomic differences. However, the contribution of lung cancer mortality to the increase in the socioeconomic differences was relatively small among both men and women. In contrast to earlier research reporting decreasing contribution of non-amenable causes to years of life lost [21], in our study other causes contributed clearly to increase in income group differences. Our results are in line with earlier studies reporting differences in years of life lost [21] and the contribution of mortality amendable to health care to socioeconomic differences in life expectancy [18].

A strength of our study was the ability to utilise an individually linked indicator of socioeconomic position. The income data used in the study come from the registers of tax administration and the Social Insurance Institution, and are thus not vulnerable to reporting bias and missing information. The use of family income instead of individual income is likely to produce more robust results since family income is influenced less by potential income loss due to ill health and therefore less sensitive to reverse causation. We also performed a sensitivity analysis for 2006-07 using income data from 2002-03. Only minor differences were detected in life expectancy figures or their patterns. The longitudinal nature of our data enabled us to analyse changes in socioeconomic differences in life expectancy. The Finnish Cause of Death statistics are considered valid and reliable by international standards [39]. Furthermore, the rate of confirmation of the diagnosis by autopsy in Finland is high compared to many other countries (30% for all deaths and about 60% of those of working age) [40].

We had two indicators of mortality amenable through health policy measures, alcohol related and lung cancer mortality. Our measure of alcohol related mortality is based on a standard classification by Statistics Finland [29] and takes into account not only alcohol poisonings but also diseases brought up by excess consumption. We used lung cancer as an indicator of smoking related mortality, which clearly leads to an underestimation. WHO estimates concerning health risks suggest that lung cancer mortality covers 30 to 40 per cent of mortality attributable to smoking in most Western European countries [41]. While smoking is the main cause of lung cancer, some of the smoking related mortality is mediated through IHD and some, like COPD was in the current study classified to mortality amenable through health care. Another limitation of our study is that analysis of mortality amenable through policy actions using mortality data can only yield indirect evidence of their potential effect, and socioeconomic differences in mortality are likely to arise from complex interplay of different factors affecting the social and economic circumstances and health of individuals. Mortality amenable to health care has, in the last decades, been increasingly used as an indirect indicator for functioning of health care, but it has known limitations [10]. The indicator does not take into account disease incidence or prevalence or whether deaths occur due to shortcomings in access and quality of care or avoidance and non-compliance on part of the patient.

In addition to total life expectancy at age 35, we also analysed partial life expectancy from age 35 to 75, since mortality amenable to health care is often reported for those under the age of 75. Causes of death contributing to differences between the highest and the lowest income decile proved to be very similar. However, the analysis of only those under 75 years of age underestimates the differences in life expectancy since mortality amenable to health care contributes to years of life lost also after the age of 75. Our results suggest that the analysis of total life expectancy gives a more comprehensive picture of the contribution of amenable mortality to differences between population groups or time periods. Additional analyses showed that the age groups between 35-64 were mostly behind the decline of the life expectancy in the lowest decile among men and the stagnation among women (results not presented here). This is in accordance with previous study in Finland [4].

In line with earlier research from Finland [4], alcohol related mortality was found to be an important contributor to differences in life expectancy at age 35 and in partial life expectancy between 35 and 75 years especially among men. Among men, 82 per cent, and among women, 84 per cent of the increase in the gap in life expectancy between the highest and the lowest deciles originated from the ages 35-64 (results not presented here). Further, alcohol related mortality was the single most important contributor to the increase in socioeconomic differences from the late 1990s to 2007. These changes happened during a period of significant changes in Finnish alcohol policy. In 2004 the derogation concerning quantitative quotas for travellers’ alcohol imports from other EU countries was abolished in Finland enabling import of unlimited amounts of alcohol within the EU without further tax consequence and in the same year alcohol excise duties were decreased by, on average, a third. International research indicates that public policies affecting the price of alcohol have significant effects on alcohol consumption [42] and its health consequences [43]. Accordingly, the price reduction in Finland led to an increase of heavy drinking and heavy episodic drinking especially among men in lower socioeconomic groups [44], and to a large increase in alcohol related mortality especially among lower socioeconomic groups [45]. On the other hand, the mortality to alcohol related causes had increased markedly already before the price reduction as it shortened the life expectancy of the lowest income quintile by roughly 0.5 years among both genders from 1988-91 to 2000-04 [46]. Drawing on earlier research, it is not a surprise that alcohol related mortality had a large contribution to differences in life expectancy between the top and bottom income deciles and in changes in these differences. The contribution of alcohol related mortality to the stagnation of life expectancy in the least affluent group has earlier been reported to be especially evident when using income as the indicator of socioeconomic position instead of occupational social class or education [4]. This is probably because the lowest income decile is a smaller and more homogenous group at the bottom of the social hierarchy than either manual workers or those with basic education only.

In addition to price policy, also possible changes in the composition of the lowest income group may have an effect in the increase of the differences. Unemployment was still almost 15 per cent in the mid-1990s after the deep recession in the early 1990s, and it declined slowly after that, whereas the years 2006-07 were characterised by an economic boom. The lowest decile may therefore have been more heterogenic in the first period compared to the second. Additionally, while the income level rose during the study period in all income deciles, income inequality increased during the study period and the lowest income decile was relatively poorer towards the end of the study period [47].

Similar results concerning growing mortality differences between socioeconomic groups have been reported from, e.g., Russia [48], Estonia, Lithuania, Poland, and Hungary [49]. While these studies do not examine alcohol related mortality, the authors conclude that these differences are likely to be due to increasingly unequal social circumstances. A Scottish study [50] has also examined alcohol related mortality and the results concerning total and alcohol related mortality differences are similar to our findings. The study also found increasing differences in suicide and assault mortality as well as drug related mortality suggesting that the differences are underpinned by unequal social circumstances.

Whereas the direct contribution of health care to socioeconomic differences and changes in them was according to our results relatively modest especially among men, it must be borne in mind that part of ischaemic heart disease mortality should also be attributed to health care. International studies have estimated the impact of treatment and population risk factor reductions to the decline in ischaemic heart disease mortality combining register data on morbidity and mortality and health care use and survey and audit data on risk factors among both men and women. Studies from England and Wales [31], Scotland [32], Australia [51], Ireland [52] and Canada [33] have attributed 40-44 per cent of the decline to treatment effects and 48-58 per cent to population risk factor reductions. In Finland, a similar estimation attributed 23 per cent of the reduction to treatment effects and 53-72 per cent to risk factor reduction between 1982 and 1997 [34]. In international comparison the proportion attributed to risk factor reduction is unusually large. However, the Finnish estimate comes from 1997. Developments in coronary care since then may have increased the effect of health care to the decline in coronary mortality and to increasing socioeconomic differences in it. Nevertheless more research is needed to ascertain whether coronary care has improved more in more affluent groups.

Our results concerning the role of health care are in line with earlier findings from Finland and elsewhere reporting differential access of both primary care [11] and access to and quality of specialist care [12-15] by socioeconomic position. While health care could be influential in decreasing socioeconomic health differences, our results suggest that the three tier system of ambulatory care in Finland may not have been successful in decreasing health and mortality differences but has rather contributed to the increase of these differences. Mortality amenable to primary care declined more rapidly in the higher rather than lower income groups, thus actually contributing to an increase of mortality inequities especially among women. More attention should be paid in improving access to and effectiveness of primary care among the less affluent in order to decrease health inequities.

## Conclusions

According to our results more than half of socioeconomic differences in life expectancy at 35 were amenable through health policy action or health care in 2006-07. Their contribution was also considerable to the increase of socioeconomic differences from 1996-97 to 2006-07. Our results underline the importance of active health policy and health care measures in tackling socioeconomic health inequalities.

## Ethics approval

This study was conducted with the approval of the ethics committee of Statistics Finland.

## Competing interests

Kristiina Manderbacka, I have the following competing interests: none declared. Riina Peltonen, I have the following competing interests: none declared. Sonja Lumme, I have the following competing interests: none declared. Ilmo Keskimäki, I have the following competing interests: I have received funding from the Academy of Finland for the study but the Academy had no involvement in its design, data collection, findings or decision to publish. I am asked to advise the Finnish Ministry of Health and Social Affairs from time to time on matters relating to health policy and services; regardless of the findings of this study, the outputs of this research would form part of that advice. Lasse Tarkiainen, I have the following competing interests: none declared. Pekka Martikainen, I have the following competing interests: I have received funding from the Academy of Finland for the study but the Academy had no involvement in its design, data collection, findings or decision to publish.

## Authors’ contributions

KM contributed to the conception and design of the study, planning of analyses, and drafted the manuscript. RP contributed to the conception and design of the study, performed the statistical analyses and took part in the revision of the manuscript for important intellectual content. SL contributed to the conception and design of the study, planning of analyses, drafting the manuscript and took part in the revision of the manuscript for important intellectual content. IK contributed to the conception and design of the study, planning of analyses and took part in the revision of the manuscript for important intellectual content. LT contributed to the conception and design of the study, planning of analyses and took part in the revision of the manuscript for important intellectual content. PM contributed to the conception and design of the study, planning of analyses, the collection of the data and took part in the revision of the manuscript for important intellectual content. All authors have read and approved the final manuscript.

## Authors’ information

KM holds PhD in sociology and works as research director in the National Research and Development Centre for Welfare and Health (THL). A main focus of her research has been equity issues in health services research. RP is MSc student in demography and she works as research assistant in Population Research Unit, Department of Social Research at University of Helsinki. She is preparing a master’s thesis on mortality amenable to health care. SL is doctoral student in public health and works as researcher in THL. She is currently finalizing her thesis concerning statistical methods to measure equity in health and health care. IK holds PhD in public health and works as research professor in THL. A main focus of his research has been equity issues in health services research. Currently he leads a project examining mortality amenable to health care. He also works part time (20%) as professor of social and health policy at University of Tampere, School of Health Sciences. LT is doctoral student in demography and works as researcher in Population Research Unit, Department of Social Research at University of Helsinki. He is preparing his thesis on socioeconomic differences in mortality. PM holds PhD in population studies and works as professor of demography in Department of Social Research at University of Helsinki. A main focus of his research has been socioeconomic differences in mortality. Currently he leads a project on the subject.

## Pre-publication history

The pre-publication history for this paper can be accessed here:

http://www.biomedcentral.com/1471-2458/13/812/prepub

## Supplementary Material

Additional file 1 **List of causes of death considered amenable to health care or health policy and the corresponding ICD-10 codes.** The file contains all the causes of death considered amenable to health care and health policy (alcohol related and lung cancer mortality) and those considered amenable by both (IHD). Click here for file
