# Supplementary material for: The contribution of health policy and care to income differences in life expectancy – a register based cohort study
Source: BMC Public Health. 2013 Sep 8;13:812. doi: 10.1186/1471-2458-13-812 (PMC3846484; doi:10.1186/1471-2458-13-812)
Supplement: Additional file 1 — List of causes of death considered amenable to health care or health policy and the corresponding ICD-10 codes. The file contains all the causes of death considered amenable to health care and health policy (alcohol related and lung cancer mortality) and those considered amenable by both (IHD). [file 1471-2458-13-812-S1.pdf]

Additional file 1. List of causes of death considered amenable to health care or health policy and the corresponding ICD-10 codes

Place of intervention	Cause of death	Age[1]	ICD-10
PRIMARY HEALTH CARE			
Primary prevention	Diphtheria, Tetanus, Poliomyelitis, and Varicella	1–74	A35–36, A80, B01
	Rubella	1–74	B06
	Scarlatina	1–74	A38
	Meningococcus	1–74	A39
	Erysipelas	1–74	A46
	Legionellosis	1–74	A48.1
	Malaria	1–74	B50–54
	Streptococcal pharyngitis	1–74	J02.0
	Cellulitis	1–74	L03
Early detection and treatment	Tuberculosis	1–74	A15–A19, B90
	Malignant neoplasm of colon and rectum	1–74	C18–21
	Melanoma of skin	1–74	C43
	Malignant neoplasm of skin	1–74	C44
	Malignant neoplasm of breast	1–74	C50
	Malignant neoplasm of cervix uteri	1–74	C53
	Malignant neoplasm of cervix uteri and body of uterus	1–44	C54–55
	Malignant neoplasm of bladder	1–74	C67
	Benign tumors	1–74	D10–36
	Hypertensive disease	1–74	I10–13, I15
	Cerebrovascular disease	1–74	I60–69
Improved treatment and medical care	Diseases of the thyroid	1–74	E00–07
	Diabetes mellitus	1–49	E10–14
	Epilepsy[2]	1–74	G40–41
	Asthma	15–49	J45–46
	COPD	15–49	J40–44
SPECIALIZED HEALTH CARE	Septicaemia	1–74	A40–41
	Malignant neoplasm of testis	1–74	C62
	Hodgkin’s disease	1–74	C81
	Leukaemia	1–44	C91–95
	Rheumatic and other valvular heart disease	1–74	I01–09
	Influenza	1–74	J09–11
	Pneumonia	1–74	J12–18

	Peptic ulcer	1–74	K25–28
	Appendicitis	1–74	K35–38
	Abdominal hernia	1–74	K40–46
	Cholelithiasis and cholecystitis	1–74	K80–81
	Nephritis, nephrosis, and nephropathy	1–74	N00–N09, N17–N19, N25–N27
	Obstructive uropathy and prostatic hyperplasia	1–74	N13, N20–N21, N35, N40
	Maternal death	All	O00–O99
	Congenital cardiovascular anomalies	1–74	Q20–Q28
HEALTH POLICY			
	Mental and behavioural disorders due to use of alcohol	All	F10
	Degeneration of nervous system due to alcohol	All	G31.2
	Alcohol related epilepsy [2]	All	G40.51
	Alcoholic polyneuropathy	All	G62.1
	Alcoholic myopathy	All	G72.1
	Alcoholic cardiomyopathy	All	I42.6
	Alcoholic gastritis	All	K29.2
	Alcoholic liver disease	All	K70
	Alcohol-induced acute pancreatitis[3]	All	K85.2
	Alcohol-induced chronic pancreatitis	All	K86.0
	Maternal care for (suspected) damage to foetus from alcohol	All	O35.4
	Accidental poisoning by and exposure to alcohol	All	X45
	Lung cancer	All	C32-C34
HEALTH CARE AND POLICY			
	Ischaemic heart disease	All	I20-I25

[1] In our study, deaths of people younger than 35 were excluded from the analyses.
[2] Alcohol related epilepsy was included in deaths amenable through health policy and not mortality amenable through health care.
[3] Only in 2007 (new code in ICD-10 classification).
